# Cross species reproducibility of MRI radiomics features enables intervertebral disc degeneration assessment in experimental monkeys

**DOI:** 10.1038/s41598-025-29167-z

**Published:** 2025-11-28

**Authors:** Jianmin Wang, Lei Guo, Jianfeng Li, Xiaodong Cao, Wei Du, Jiaxiang Zhou, Haizhen Li, Junhong Li, Zhengya Zhu, Tao Tang, Xianlong Li, Zhiyu Zhou, Zhiguo Liu, Yongming Xi, Manman Gao

**Affiliations:** 1 Department of Spine Surgery, Yantaishan Hospital, Binzhou Medical University, Yantai, 264003 Shandong China; 2 Innovation Platform of Regeneration and Repair of Spinal Cord and Nerve injury, Department of Orthopaedic Surgery, The Seventh Affiliated Hospital, Sun Yat-sen University, Shenzhen, 518107 Guangdong China; 3 Department of Neurology, Yantaishan Hospital, Binzhou Medical University, Yantai, 264003 Shandong China; 4 School of Materials Science and Engineering, South China University of Technology, Guangzhou, 510641 Guangdong China; 5 Department of General Practice, The Seventh Affiliated Hospital, Sun Yat-sen University, Shenzhen, 518107 Guangdong China; 6 Department of Orthopedics and Trauma, Affiliated Hospital of Yunnan University, Kunming, 650032 Yunnan China; 7 Department of Orthopaedic Surgery, The Affiliated Hospital of Xuzhou Medical University, Xuzhou, 221006 Jiangsu Province China; 8 Department of Orthopaedics, The Second Affiliated Hospital of Anhui Medical University, Hefei, 230601 Anhui China; 9 Guangdong Provincial Key Laboratory of Orthopedics and Traumatology, The First Affiliated Hospital of Sun Yat-sen University, Guangzhou, 510080 Guangdong China; 10 Department of Neurosurgery, Central Hospital Affiliated to Shandong First Medical University, No.105 Jiefang Road, Lixia District, Jinan, 250012 Shandong China; 11 Department of Orthopaedic Surgery, The Affiliated Hospital of Qingdao University, No.59 Haier Rd, Laoshan District, Qingdao, 266000 Shandong China; 12 Orthopedic Research Institute, Fuzhou Second Hospital, No.47 Shangteng Road, Fuzhou, 350007 Fujian China; 13 Department of Pediatric Orthopaedic, Fuzhou Second Hospital, Fuzhou, 350007 Fujian China; 14 Department of Neurosurgery, Jinan Zhangqiu District Hospital of Traditional Chinese Medicine, Jinan, 250200 Shandong China

**Keywords:** Radiomics, Radiomics model, Generalizability, Machine learning, Experimental monkey, Machine learning, Diagnostic markers, Animal physiology

## Abstract

Experimental monkeys serve as a bridge between basic research and clinical medicine. Accurately assessing the degree of intervertebral disc degeneration (IVDD) in experimental monkeys is crucial for further intervertebral disc related research in these animals. Radiomics promises significant enhancement in quantitative diagnostic precision for IVDD, while the cornerstone of constructing robust and efficient radiomics models (RMs) relies on access to large-scale sample data. In experimental monkey research, however, ethical restrictions and resource constraints typically limit sample sizes. This study addresses this challenge by comparing and analyzing the generalizability of intervertebral disc MRI-based radiomics models between humans and experimental monkeys. The findings reveal that 12.30% (438/3562) of the radiomics features demonstrate high reproducibility between the two species. Leveraging the sufficient human dataset, we built RMs and employed the experimental monkey dataset as a training set to validate the cross-species generalizability of these models. Notably, in the test phase, models constructed based on the inter-species reproducible features achieved AUC values ranging from 0.82 to 0.92, indicative of promising diagnostic performance. This study emphasizes the advantages of leveraging human data for the construction of RMs under conditions of constrained experimental monkey research. We innovatively propose and validate the potential for cross-species application of RMs. This study furnishes strong theoretical underpinnings and practical foundations for the broader application of radiomics in cross-species disease research.

**Supplementary Information:**

The online version contains supplementary material available at 10.1038/s41598-025-29167-z.

## Introduction

Experimental monkey species exhibit astonishing similarities to humans in locomotor behavior patterns, cell composition of the intervertebral disc (IVD), and the progression of intervertebral disc degeneration (IVDD), making them an ideal alternative animal model for studying human IVDD^1^. Furthermore, the experimental monkey model is the final step in preclinical development of drugs and vaccines, greatly improving the reliability of research results and providing a solid foundation for the eventual clinical translation. Experimental monkeys play an important role in basic and translational biomedical research, serving as a bridge between basic research and clinical medicine.

In the research of experimental monkey IVDD, diagnostic errors in the degree of IVDD could seriously affect subsequent related research. Consequently, accurate assessment of the degree of IVDD holds great significance for in-depth research into the pathophysiology and therapeutic interventions of the condition. At present, the degree of IVDD is often determined using the Pfirrmann grading^2^. However, it has considerable subjectivity and inability to distinguish minor differences in IVDD^2^. Meanwhile, when graded by different observers, differences of opinion could also arise^3^. The pursuit of innovative techniques for accurate assessment of the degree of IVDD holds considerable significance in advancing the research and improving therapeutic strategies targeting IVDD.

As a significant innovation in medical imaging analysis technology in recent years, radiomics could quantitatively extract and analyze high-throughput radiomics features (RFs) from medical images, providing richer and more accurate information to assist in clinical disease diagnosis and treatment outcome prediction^4^. Radiomics has shown great value in applications such as diseases diagnosis^5^, prediction of clinical treatment outcomes^6–8^, assessment of the pathological heterogeneity of whole tumor tissues^9,10^, and gene expression prediction^11^. Therefore, radiomics technology should be adopted to extract high-throughput RFs from IVD imaging data, with the aim of more accurately assessing the degree of IVDD based on these high-throughput RFs.

When constructing radiomics models (RMs), a common challenge is the limited sample size, which reduces the robustness and reliability of RMs. Large sample size could enhance the performance of RMs. In the study of experimental monkeys, due to ethical and resource constraints, the number of precious animals is limited, which cannot meet the sample size requirements of radiomics for study subjects, while these data could be obtained more easily in humans. There is a large amount of human IVD data available in clinical practice for constructing RMs. Therefore, we propose a hypothesis that RMs could be constructed based on human IVD data and then applied in experimental monkeys. However, it is unclear whether the RFs of the IVDs between experimental monkey and humans are reproducible and whether the resulting RMs could be applied across species. Cross-species application of the IVD RM has not previously been well established in the literature.

**Fig. 1:**
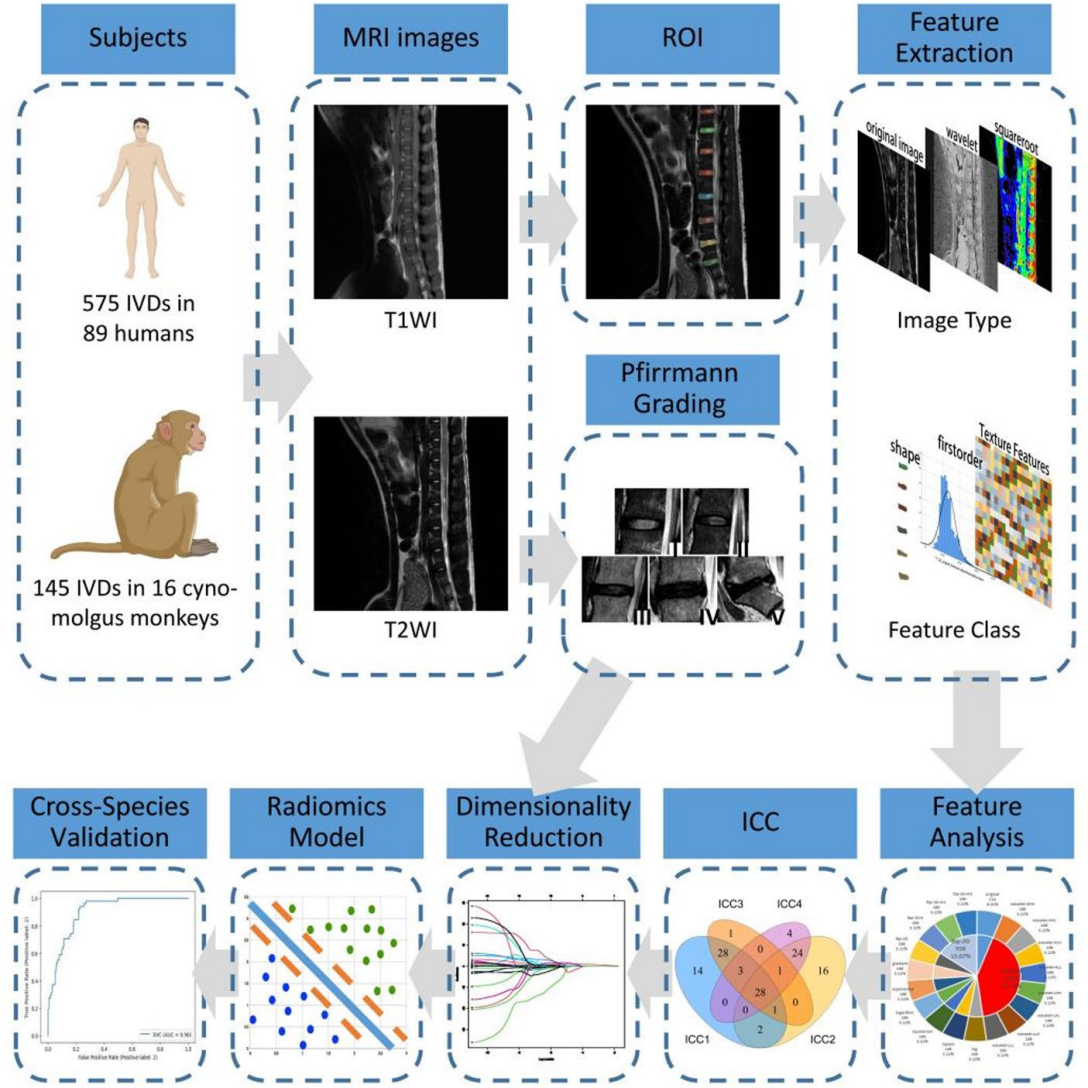
A flowchart of the study process. MRI data of 720 IVDs are obtained from humans and experimental monkeys. Extract radiomics features, and then *t*-test combined with LASSO method is used to select features with reproducibility between humans and experimental monkeys. Analyze the distribution characteristics of reproducible features and compare the differences in ICC values between humans and experimental monkeys. Radiomics models are constructed based on the human dataset and the dataset from the experimental monkey is used as a test set to verify the generalizability of the model between species. ICC = intraclass correlation coefficient, IVD = intervertebral disc, ROI = region of interest, T1WI = T1-weighted imaging, T2WI = T2-weighted imaging.

In the present study, we analyzed the reproducibility of RFs in humans and cynomolgus monkeys using lumbar and lower thoracic IVDs as subjects. We also used the human dataset as a training set to construct RMs to predict the degree of disc degeneration and validated the interspecies generality of the RMs with the experimental monkey dataset. Our objective is to develop a methodology that compensates for the limitation in sample size when constructing RMs, thereby enabling accurate assessment of the degree of IVDD in experimental monkeys. This will pave the way for further experimentation and research concerning IVDD in experimental monkey model. A diagram of this workflow is illustrated in Fig. 1.

## Materials and methods

### Study subjects

This study included a prospective dataset from cynomolgus monkeys and a retrospective dataset from human volunteers. The experimental protocol involving cynomolgus monkeys was reviewed and approved by the Ethics Committee of the Institute of Zoology, Guangdong Academy of Sciences (Approval No. G2Z20210103). All animal experiments were conducted strictly in accordance with the ARRIVE guidelines (https://arriveguidelines.org) and relevant national regulations on laboratory animal welfare. The study involving human volunteers was approved by the Ethics Committee and Institutional Review Board of the First Affiliated Hospital of Sun Yat-sen University (Approval No. 2008-55). The study was conducted in accordance with the Declaration of Helsinki, and written informed consent was obtained from all human subjects before enrollment.

Experimental animals were purchased from Guangzhou Topgene Biotechnology Inc., with complete birth records, research-related documentation, and quarantine certificates available. All procedures including the housing and care of the animals were conducted at the Institute of Zoology, Guangdong Academy of Sciences, while the MRI scans were performed at Foresea Life Insurance Guangzhou General Hospital. The experimental monkeys completed lumbar MRI in 2021. The human study subjects were volunteers who underwent MRI examinations of the lumbar spine at the First Affiliated Hospital of Sun Yat-sen University from July 2009 to November 2010. The inclusion criteria were as follows: ① Human volunteers who volunteer for research. ② Study subjects had no history of spinal surgery, trauma, and experimental spinal research. The exclusion criteria were as follows: Study subjects with poor image quality.

### MRI protocol

**Fig. 2:**
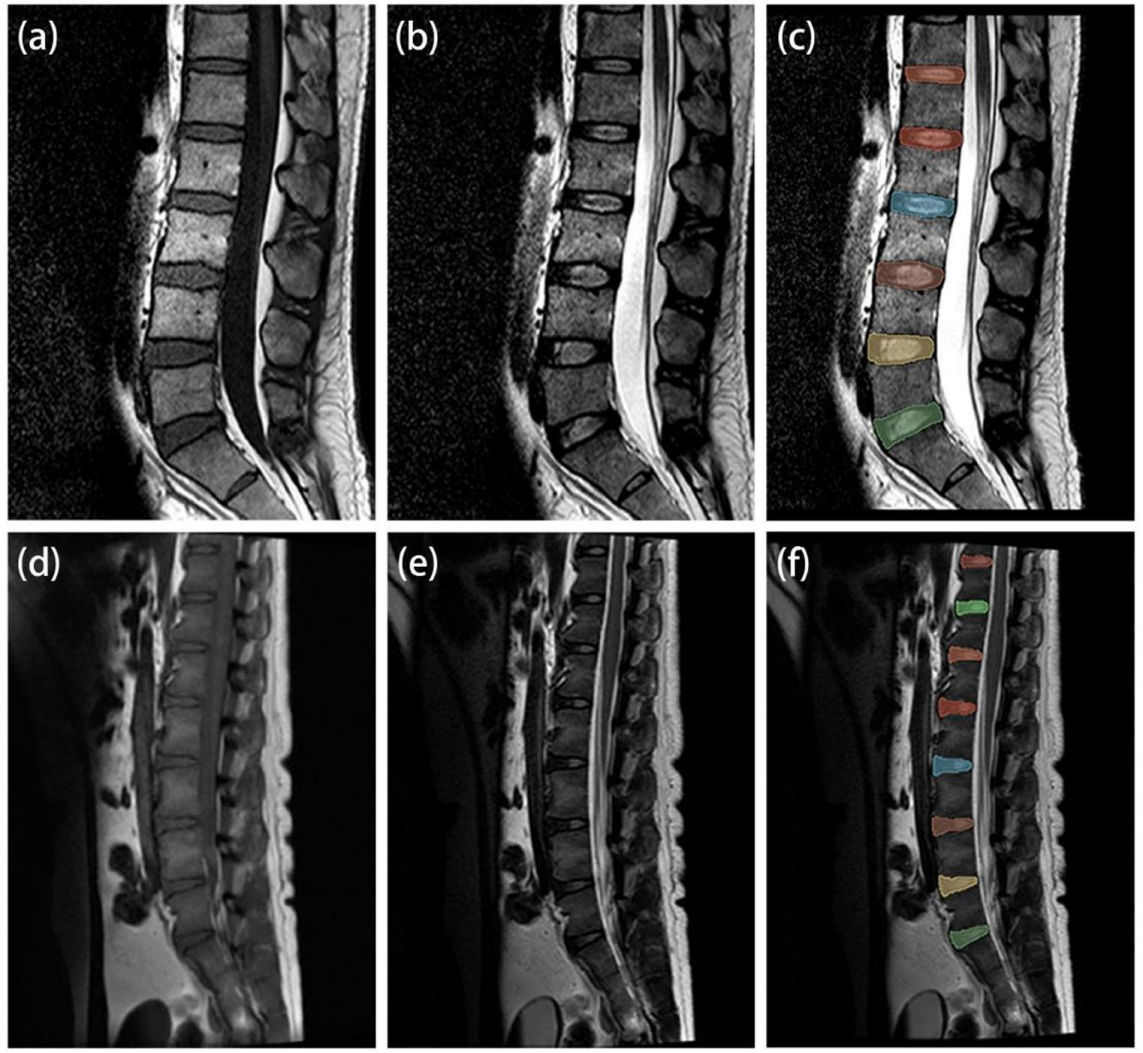
MRI cases of human and experimental monkey. (**a**–**c**) Images in a 32-year-old man human. (**d**–**f**) Images in a 20-year-old male experimental monkey. (**a**) and (**d**) T1-weighted imaging. (**b**) and (**e**) T2-weighted imaging. (**c**) and (**f**) segmented intervertebral disc regions of interest.

Human MRI data were acquired using a 1.5-Tesla MRI scanner (Philips, United States). The experimental monkey MRIs were all performed using a 3.0-Tesla MRI scanner (General Electric Company, United States). All scans were performed in the supine position. The MRI protocol for the lumbar spine consisted of a sagittal T1WI sequence and T2WI sequence (Table 1; Fig. 2). The experimental monkey MRIs were preceded by anesthesia delivered by an experienced veterinarian who was also responsible for animal care. Prior to MRI scanning, the experimental monkeys were fasted for 8 h and then anesthetized via intramuscular injection of tiletamine hydrochloride and zolazepam hydrochloride (Zoletil^®^ 50, Virbac, France) at a dose of 8–10 mg/kg (concentration: 50 mg/ml).

**Table 1:** Parameters for mr imaging of humans and experimental monkeys.

Parameter	Experimental monkey		Human	
	T1WI	T2WI	T1WI	T2WI
Pulse sequence	FSE	FSE	Spin echo	Spin echo
Repetition time (ms)	500	2000	448	3228
Echo time (ms)	8	102	8.0	100
Field of view (mm)	20 × 20	20 × 20	250 × 250	250 × 250
Pixel bandwidth (Hz)	390.6	325.5	320	348
Voxel size (mm)	0.8 × 0.8	0.8 × 0.8	0.9 × 1.25	0.9 × 1.25
Slice thickness (mm)	3	3	3	3
Interslice gap (mm)	0.6	0.6	0.3	0.3
Number of slices	7	7	15	15
Turbo factor	3	16	4	45
Acquisition time	70	100	208	160

T1WI = T1-weighted imaging, T2WI = T2-weighted imaging.

### Pfirrmann grading

The degree of disc degeneration was determined according to the criteria described by Pfirrmann et al.^2^. The grading process was performed independently by 2 experts (Zhiyu Z., and Jianmin W., with 25, 8 years of clinical work experience, respectively) performing 2 analyses each at least 1 week apart. Inconsistencies in the disc grading results were resolved later by discussion among the 2 experts. In the subsequent radiomics analysis, we considered grades Ⅰ-Ⅱ to represent normal discs and marked them as 0 and grades Ⅲ-Ⅴ to represent degenerated discs and marked them as 1.

### Image data preprocessing and image segmentation

We used the N4ITK Bias Field Correction module (https://www.slicer.org/wiki/Documentation/Nightly/Modules/N4ITKBiasFieldCorrection)^12^ in 3D Slicer software (https://www.slicer.org/, version 4.11.20210226) to perform bias fields corrections.

The regions of interest (ROIs) were manually segmented in 3D Slicer software in the median sagittal plane of the target disc and included the entire nucleus pulposus, annulus fibrosus, and endplate (Fig. 2). The ROIs were initially segmented on the T2WI and subsequently replicated on the T1WI by two specialists (Zhiyu Z., J.W.). One of the specialists (Zhiyu Z.) performed 2 analyses, at least 1 week apart; the ROIs outlined the second time were selected for the RF analysis and modeling studies.

### Extraction of radiomics features

PyRadiomics (https://pyradiomics.readthedocs.io/en/latest/index.html, version 3.0.1)^13^, an Image Biomarker Standardization Initiative (IBSI)^14^ guideline-compliant program, was used to extract the RFs on the images from both the T1WI and T2WI sequences. The images were normalized before feature extraction^15^, then resampled (resampled voxel size set to 1,1,1) with a binWidth of 25. ‘sitk.sitkBSpline’ was used as an interpolator. All features based on the original image and derived images were extracted. This collection was defined as feature set A.

### Screening of reproducible radiomics features between humans and experimental monkeys

First, we used the independent-samples *t*-test to screen for reproducible features between humans and experimental monkeys. Features with *p* < 0.05 were considered to have significant species differences and were excluded, retaining only those with no statistically significant interspecies differences (*p* ≥ 0.05). We further screened for reproducible RFs between species using least absolute shrinkage and selection operator (LASSO) regression, where the predictors were the remaining RFs after *t*-test filtering, and the response variable was the species label (human vs. experimental monkey). LASSO regression imposes an L1 penalty to force some regression coefficients to zero, which effectively selects features with little or no contribution to distinguishing between species. In this study, we treated features with regression coefficients equal to 0 as those that contributed less to interspecies differences, meaning that they are reproducible features across species. Reproducible features between the species screened with the combination of the independent-samples *t-*test and LASSO to form feature set B.

### Intraobserver and interobserver agreement

The intraclass correlation coefficient (ICC) assessed RFs reproducibility from manual ROI segmentation, with “observers” defined per Sect. 2.4: two specialists (Zhiyu Z. and J.W.), with Zhiyu Z. performing a second independent segmentation at least one week apart.

Using the Pingouin package (version 0.5.1) with a two-way random effects model (absolute agreement), we calculated: ① intraobserver ICC (Zhiyu Z.’s two segmentations); ② interobserver ICC (Zhiyu Z.’s second segmentation and J.W.’s). Features with ICC >0.75 were considered reproducible^16^.

These features from feature set A and feature set B formed feature set A1 and feature set B1, respectively.

### Construction of the radiomics models and validation of model performance across species

First, the RFs were standardized using z-scores. Mutual information (MI) and LASSO regression were used for dimensionality reduction. MI: Python’s sklearn.feature_selection.mutual_info_classif calculated MI between each feature (A1/B1) and IVDD label (healthy vs. degenerated). Features with MI > 0.1 were retained. LASSO: R’s glmnet package was used. Optimal λ was selected via 5-fold cross-validation (Supplementary Fig. 3); features with non-zero coefficients were retained. In the LASSO dimensionality reduction process, the predictors were the features from feature sets A1 and B1 (with intra- and interobserver ICC > 0.75), and the response variable was the IVDD classification label (healthy vs. degenerated) based on Pfirrmann grading. The features retained after dimensionality reduction from feature set A1 and feature set B1 formed feature set A2 and feature set B2, respectively. We used these reduced feature sets to construct models to improve robustness and generalization ability.

To verify whether a RM could be applied with similar efficacy in both humans and experimental monkeys, we constructed RMs based on feature set A2, and feature set B2 of humans. We used the human data as a training set to construct RMs to assist in evaluating the degree of IVDD using Support Vector Machine (SVM), Decision Tree Classifier, Random Forest Classifier, Logistic Regression, and Naive Bayes Classifier, respectively. Then, the experimental monkey data were used as the test set to verify the generalizability of the RMs across species.

Due to class imbalance in the training set (human data), we used SMOTE (via imblearn.over_sampling.SMOTE) for oversampling. Key implementation details: Restricted to the training set to prevent data leakage. Performed after feature standardization and dimensionality reduction. Synthetic minority class samples were generated to balance the class distribution at a 1:1 ratio.

### Statistical analysis

IBM SPSS Statistics for Windows v.26 (SPSS, Chicago), Python (version-3.7.13), and R (version-4.1.1) were used to perform the statistical analyses. The glmnet R package (version-4.1. 4) was used to perform LASSO analyses. Differences between groups were assessed by the *t*-test (scipy package version 1.7.3) or chi square test. For all analyses, *p* < 0.05 was considered significant. Values are expressed as the mean ± standard deviation (SD). Model performance was evaluated by receiver operating characteristic (ROC) curve.

## Results

### Participant and disc characteristics

The characteristics of the 720 enrolled IVDs are shown in Table 2. In humans, a total of 90 volunteers underwent MRI and 1 case was excluded because the poor image quality. A total of 89 human volunteers (61 men, 28 women) with a mean age of 31.91 years ± 6.62 (SD) were included. MRI data of 575 IVDs were obtained from human volunteers. The Pfirrmann grading distribution of these IVDs was as follows: grade I-II: 436, grade III-V: 139. Sixteen experimental monkeys completed MRI and were included, with no excluded cases. The enrolled experimental animals had a mean age of 11.81 years ± 4.42 (SD) and a mean body weight was 7.94 kg ± 1.91 (SD). All experimental monkeys were males. MRI data of 145 IVDs were obtained from experimental monkeys. The Pfirrmann grading distribution of these IVDs was as follows: grade I-II: 100, grade III-V: 45.

**Table 2:** Participant characteristics.

Characteristic	Total	Training set	Test set	*p* value
Number of participants	105	89	16	
Number of IVDs	720	575	145	
Pfirrmann grades				0.091
I-II	536	436	100	
III-V	184	139	45	
Age (y)*	33.91 ± 9.80	31.91 ± 6.62	41.83 ± 15.12^#^	<0.001^#^
Sex				<0.001
M	538	393	145	
F	182	182	0	
Segments				<0.001
T8/9	1	-	1	
T9/10	1	-	1	
T10/11	3	1	2	
T11/12	57	44	13	
T12/L1	105	89	16	
L1/2	105	89	16	
L2/3	105	89	16	
L3/4	105	89	16	
L4/5	105	89	16	
L5/6 ^&^	101	85	16	
L6/7	16	-	16	
L7/S1	16	-	16	

(1) All data are the number of intervertebral discs or participants, except for age. (2) *: Data are means ± standard deviations. (3) #: The age of experimental monkeys has been converted into the age equivalent to that of humans in a ratio of 1:3.5. (4) &: In humans, it is L5/S1. (5) IVD: intervertebral disc.

### Analysis of radiomics features

**Fig. 3:**
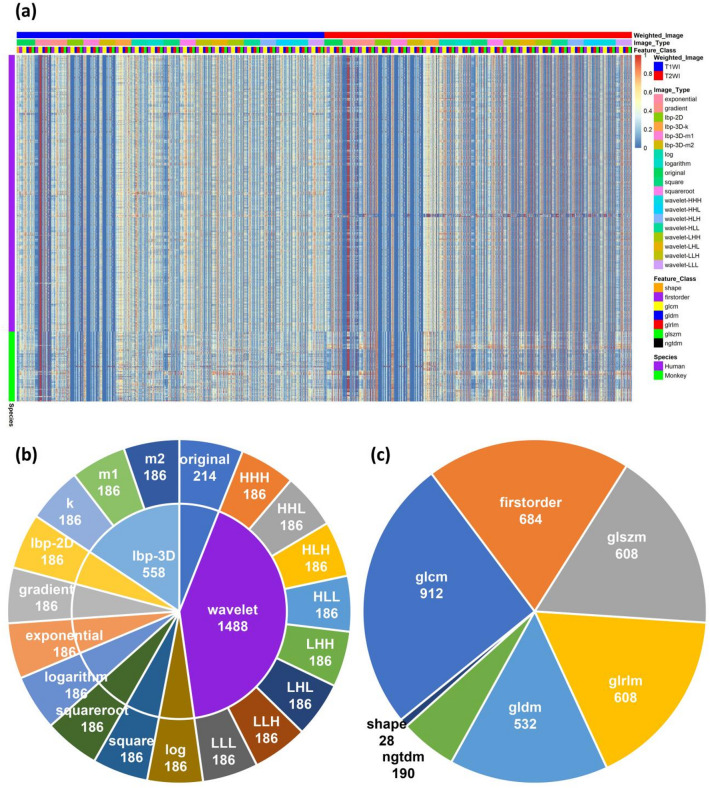
Distribution of radiomics features. (**a**) Heatmap showing all 3562 radiomics features for the 720 human and experimental monkey IVDs. (**b**) The radiomics features are distributed across 10 major and 19 minor Image Types. (**c**) The radiomics features are distributed into seven Feature Classes. (*n* = 3562) IVD = intervertebral disc, T1WI = T1-weighted imaging, T2WI = T2-weighted imaging.

In this study, 3562 features (feature set A, total number from both T1WI and T2WI sequences: 1781 from T1WI and 1781 from T2WI) were extracted from the MR images of 575 human discs and 145 experimental monkey discs (Fig. 3a). the distribution of these features across major Image Types and Feature Classes did not show significant interspecies differences. The number of features extracted was the same for both the T1-weighted imaging (T1WI) and T2-weighted imaging (T2WI) sequences (Supplementary Fig. 1a). The 3562 features were distributed in 10 Image Types, including original image and 9 derived images (Fig. 3b). The 10 Image Types were: original, wavelet, log, square, squareroot, logarithm, exponential, gradient, LocalBinaryPattern2D (lbp-2D), and LocalBinaryPattern3D (lbp-3D). The wavelet Image Type also included 8 decompositions (HHH, HHL, HLH, HLL, LHH, LHL, LLH, LLL), while the lbp-3D Image Type included 3 parameterized variants (k, m1, m2). Figure 3b shows the number of features in each Image Type. The features extracted by Pyradiomics were distributed into 7 Feature Classes, namely, First Order Features (firstorder), Shape Features, Gray Level Co-occurrence Matrix (glcm) Features, Gray Level Size Zone Matrix (glszm) Features, Gray Level Run Length Matrix (glrlm) Features, Neighbouring Gray Tone Difference Matrix (ngtdm) Features, and Gray Level Dependence Matrix (gldm) Features. Figure 3c shows the number of features in each Feature Class. Among them, the Shape features were extracted only from the original image; the remaining Feature Class features were extracted from both the original image and derived images. Of all the Feature Classes, the smallest was the Shape class, with 28 features, and the largest was glcm, with 912 features. A total of 214 features were extracted from the original image, and their distributions were identical in both the T1WI and T2WI sequences (Supplementary Fig. 1b).

### Reproducible radiomics features between humans and experimental monkeys

**Fig. 4:**
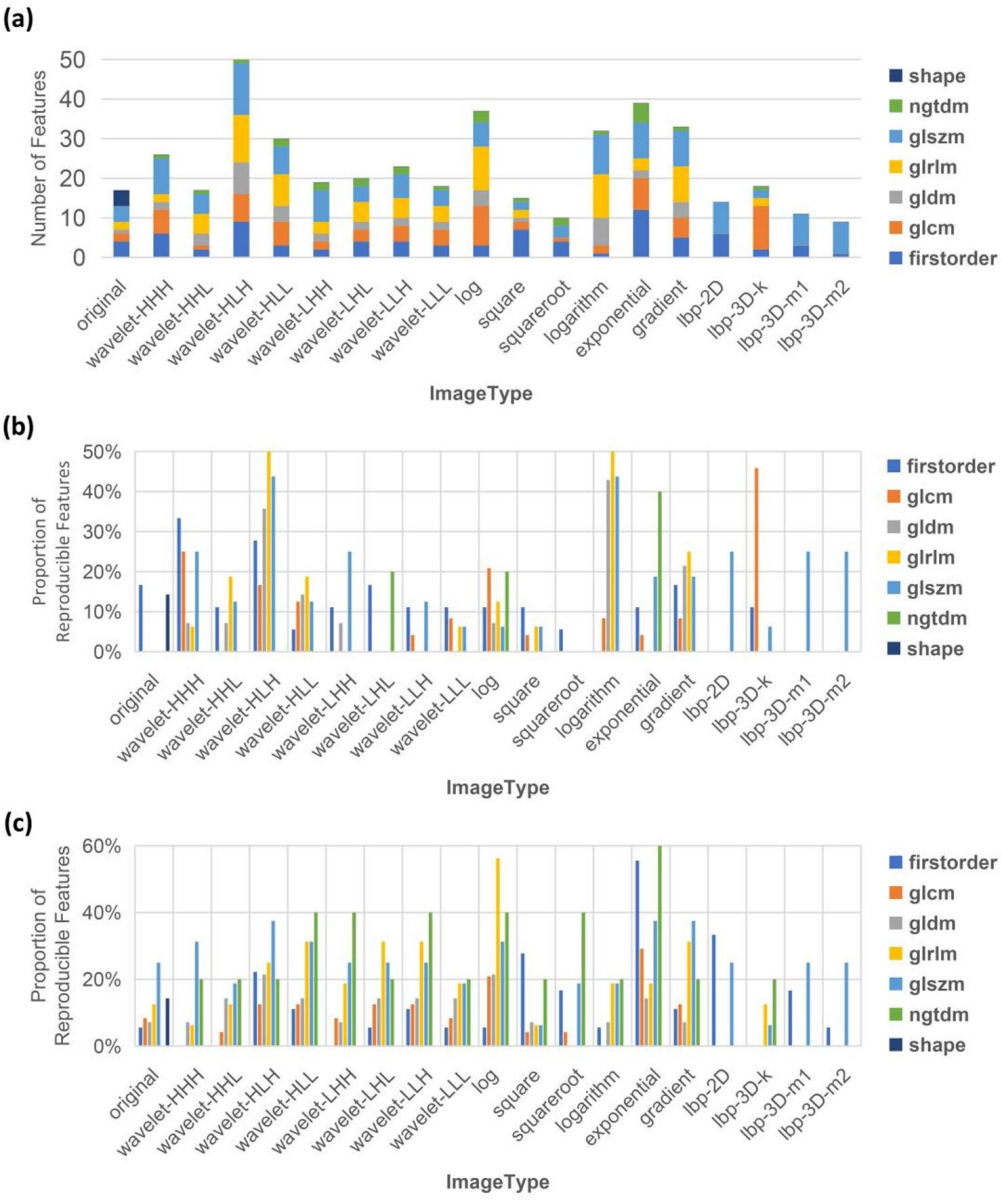
Distribution pattern of reproducible features between species screened by *t*-test combined with LASSO. (**a**) Stacked histograms show the distribution of feature set B in terms of Image Type and Feature Class. (**b-c**) Proportion of reproducible features in T1WI (**b**) and T2WI (**c**). Proportion of Reproducible Features = The number of features in each classification of feature set B / The number of features in each classification of feature set A. *n* = 438 in (**a**), *n* = 183 in (**b**), *n* = 255 in (**c**). LASSO = least absolute shrinkage and selection operator, T1WI = T1-weighted imaging, T2WI = T2-weighted imaging.

A total of 559 features were obtained after removing features with *p* < 0.05 by *t*-test. Furthermore, using least absolute shrinkage and selection operator (LASSO), 438 of these 559 features were found to be reproducible between the species. These reproducible features constitute feature set B. (Supplementary Fig. 2a). Feature set B included 183 features from T1WI and 255 from T2WI (Supplementary Fig. 2d). Of the Image Types that were represented in feature set B, the Image Types with the largest number of features was wavelet-HLH (50 of 438, 11.42%), followed by exponential (39 of 438, 8.90%) (Fig. 4a, Supplementary Fig. 2b). Among the seven Feature Classes, the Feature Class with the highest number of features was glszm (125 of 438, 28.54%), followed by glrlm (84 of 438, 19.18%) (Fig. 4a, Supplementary Fig. 2c).

In feature set B, exponential reproducible features from the T2WI and wavelet-HLH reproducible features from the T1WI accounted for the highest percentages, 33.33% (31 of 93) and 31.18% (29 of 93), respectively. Analysis of the reproducible feature percentages in terms of Feature Class shows the highest value for the glszm features from the T2WIs, at 24.67% (75 of 304) (Fig. 4b,c). At the level of original images, the detailed distribution of reproducible features across various Feature Classes is provided in Supplementary Fig. 2e.

### Intraclass correlation coefficient analysis between humans and experimental monkeys

**Fig. 5:**
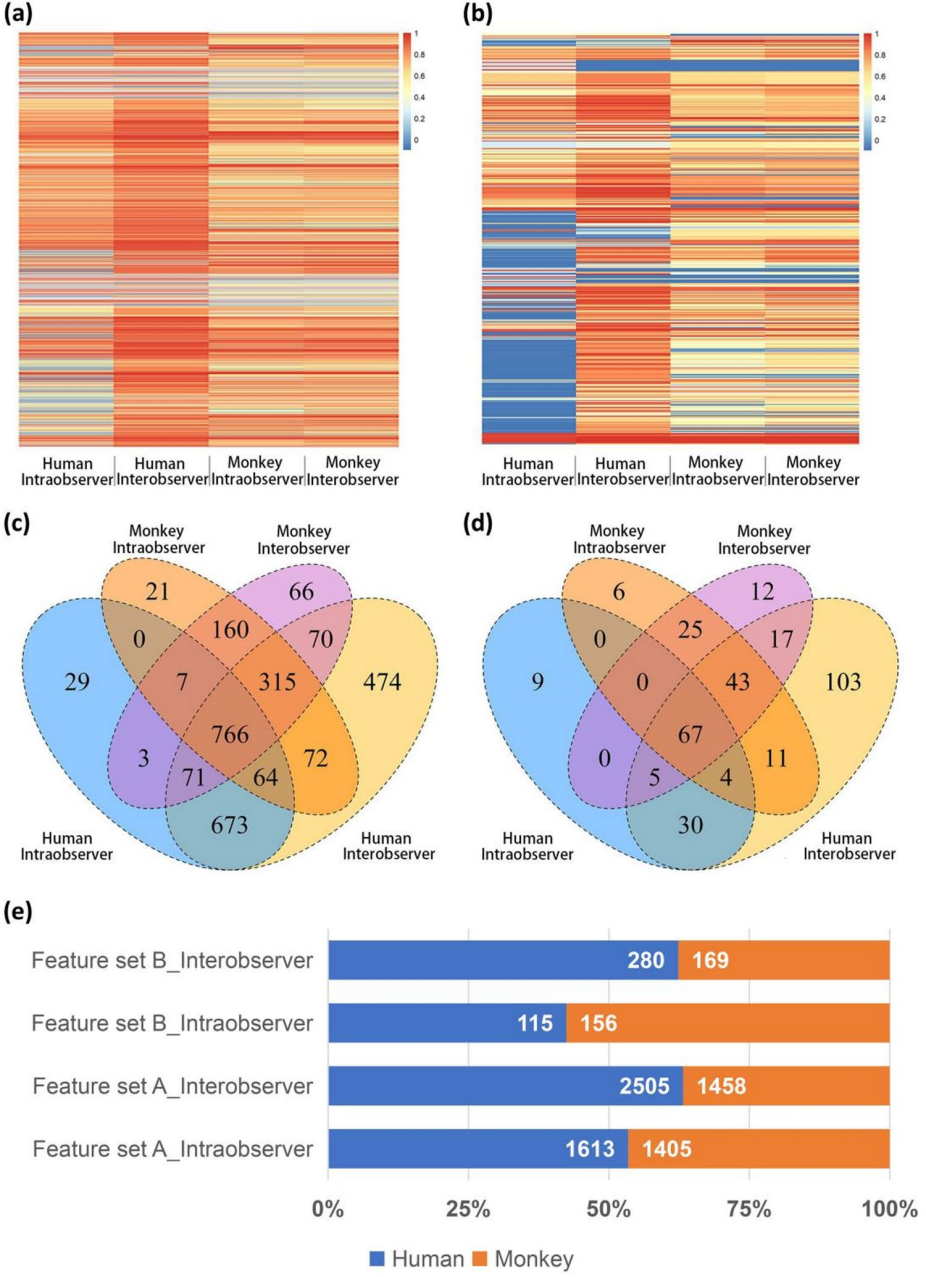
ICC analysis between humans and experimental monkeys. The ICC values of all features in feature set A (**a**) and feature set B (**b**). Venn diagrams of features with high intra- and interobserver ICCs in the feature set A (**c**) and feature set B (**d**). (**e**) Comparison of the number of features with ICC > 0.75 between humans and experimental monkeys. ICC = intraclass correlation coefficient.

Intra- and interobserver stability of the RFs between humans and experimental monkeys was determined using intraclass correlation coefficient (ICC) analysis. Figure 5a,b shows the intra- and interobserver ICC values for the 2 species in feature set A, and feature set B. In total, 766 (feature set A1), and 67 features (feature set B1) selected from each feature set that had intra- and interobserver ICCs > 0.75 for both species were used for follow-up studies (Fig. 5c,d). The proportions of human features with intra- and interobserver ICCs > 0.75 in feature set A, and feature set B were higher than the corresponding proportions of experimental monkey features, except intraobserver in feature set B (Fig. 5e).

### Effect of reproducible radiomics features screening on dimensionality reduction

**Fig. 6:**
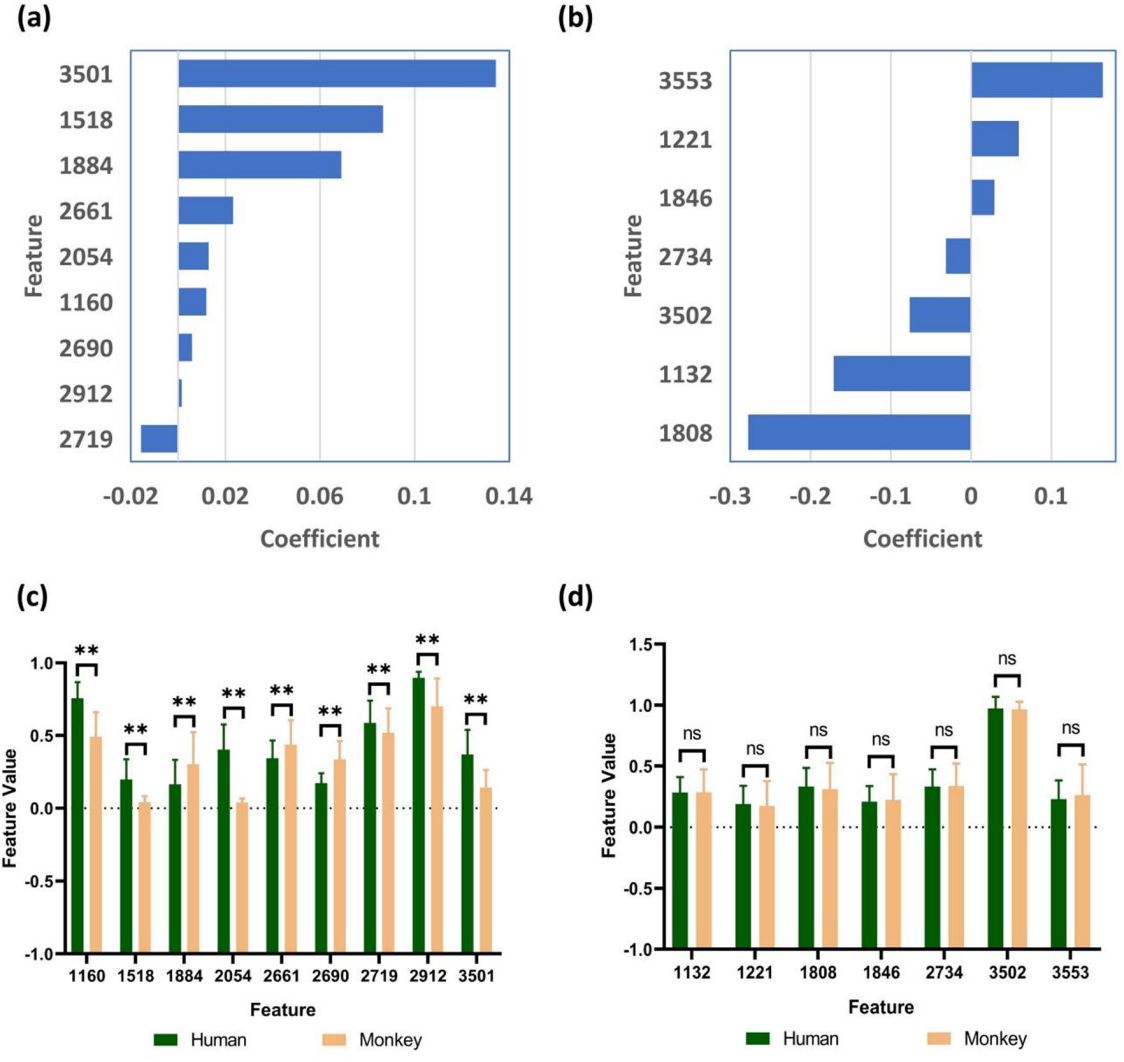
Effect of reproducible radiomics features screening on dimensionality reduction. The features and feature coefficients in feature set A2 (**a**) and feature set B2 (**b**). Comparison of feature values between species for each feature in feature set A2 (**c**) and feature set B2 (**d**). ns, *p* ≥ 0.05; ** *p* < 0.001; mean ± standard deviation (SD); *n* = 720. The feature values on the vertical axis have been standardized. The horizontal axis is the feature number. 1132: T1_wavelet-LHL_firstorder_90Percentile; 1160: T1_wavelet-LHL_glcm_Idmn; 1221: T1_wavelet-LHL_ngtdm_Complexity; 1518: T1_wavelet-HHL_firstorder_TotalEnergy; 1808: T2_original_firstorder_RobustMeanAbsoluteDeviation; 1846: T2_original_gldm_LargeDependenceHighGrayLevelEmphasis; 1884: T2_original_ngtdm_Busyness; 2054: T2_gradient_glszm_GrayLevelNonUniformity; 2661: T2_square_glcm_Idm; 2690: T2_square_glrlm_GrayLevelNonUniformityNormalized; 2719: T2_square_glszm_Zone%; 2734: T2_squareroot_firstorder_Mean; 2912: T2_wavelet-LHL_firstorder_10Percentile; 3501: T2_wavelet-LLL_glcm_Imc1; 3502: T2_wavelet-LLL_glcm_Imc2; 3553: T2_wavelet-LLL_glszm_SmallAreaHighGrayLevelEmphasis.

Nine (feature set A2), and 7 (feature set B2) RFs were obtained after filtering feature set A1, and feature set B1, respectively. The features and corresponding weights occupied of the filtered features are detailed in Fig. 6a,b. The features obtained by dimensionality reduction in two feature sets were mainly from the T2WI sequences, with percentages of 77.78% (7 of 9), and 71.43% (5 of 7), respectively (Supplementary Fig. 4a). The Image Types with the highest number of features in feature set A2 was square (3 of 9 [33.33%]), while wavelet-LHL (2 of 7 [28.57%]), wavelet-LLL (2 of 7 [28.57%]), and original (2 of 7 [28.57%]) were the largest in feature set B2 (Supplementary Fig. 4b,c). The Feature Classes with the largest number of features in feature set A2 and feature set B2 were glcm (3 of 9 [33.33%]) and firstorder (3 of 7 [42.86%]) (Supplementary Fig. 4b,c).

In feature set A2 and feature set B2, the differences in feature values were compared between humans and experimental monkeys. In feature set A2, all feature values were significantly different between humans and experimental monkeys (*p* < 0.001) (Fig. 6c). However, this difference was not statistically significant in feature set B2 (*p* ≥ 0.05) (Fig. 6d).

### Validation of radiomics models’ performance across species

The human training set had an imbalanced class distribution (436 healthy IVDs, 139 degenerated IVDs). SMOTE generated synthetic samples for the minority class, resulting in a balanced training set (436 healthy vs. 436 synthetic degenerated IVDs).

To validate the cross-species generalizability of radiomics models (RMs), we constructed two sets of models using human data as the training set: ① Models based on Feature Set A2: 9 features derived from initial features (Feature Set A1) after dimensionality reduction (no interspecies reproducibility screening). ② Models based on Feature Set B2: 7 interspecies reproducible features derived from Feature Set B1 (screened via *t*-test + LASSO in Sect. 2.6, confirmed to have no species differences).

Five algorithms were used for model construction: Support Vector Machine (SVM), Decision Tree Classifier, Random Forest Classifier, Logistic Regression, and Naive Bayes Classifier. Data from experimental monkeys were used as the test set to evaluate cross-species performance.

**Fig. 7:**
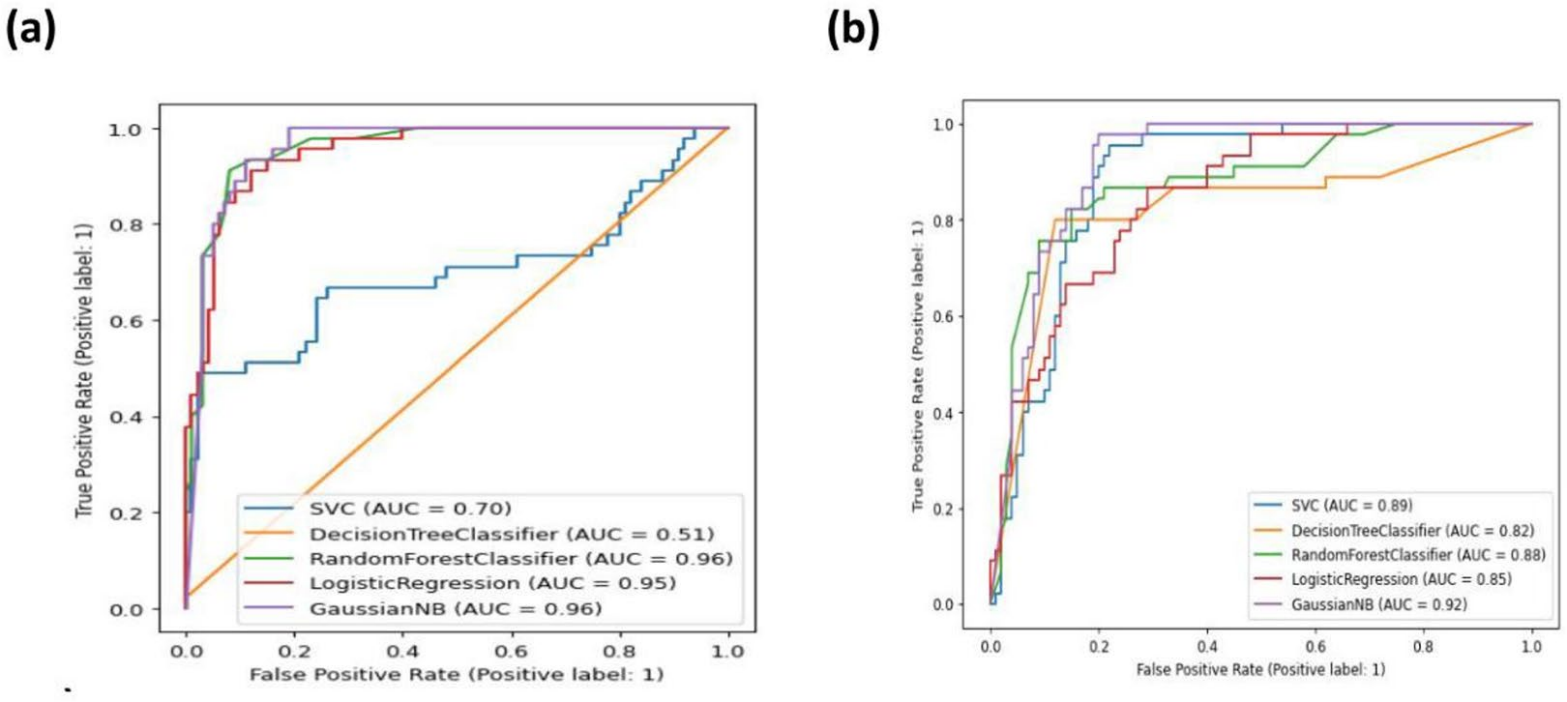
Performance measurement of radiomics models on the test set. (**a**) ROC curves for models constructed based on Feature Set A2. (**b**) ROC curves for models constructed based on Feature Set B2. AUC = area under the curve, ROC = receiver operating characteristic.

In the test set, the AUCs of the five models constructed based on feature set A2 were 0.70, 0.51, 0.96, 0.95, and 0.96, respectively, while the AUCs of the five models constructed based on feature set B2 were 0.89, 0.82, 0.88, 0.85, and 0.92, respectively (Fig. 7a,b).

Notably, although some A2 models showed high AUCs, the sensitivity of the first four A2 models (SVM, Decision Tree Classifier, Random Forest Classifier, Logistic Regression) in identifying degenerative IVDs was poor (0.36, 0.02, 0.02, 0.20, respectively; Table 3, Supplementary Fig. 5). In contrast, the sensitivity of the corresponding B2 models (trained on interspecies reproducible features) reached 0.82–0.96 (Table 3, Supplementary Fig. 5), indicating more reliable diagnostic performance for degenerated IVDs in experimental monkeys.

**Table 3:** The performance comparison of different models.

Model	Feature set	F1 score	Accuracy	Precision	Sensitivity	Specificity
Support vector machine	A2	0.508	0.786	0.889	0.356	0.980
	B2	0.748	0.800	0.614	0.956	0.730
Decision tree classifier	A2	0.043	0.697	1.000	0.022	1.000
	B2	0.667	0.745	0.561	0.822	0.710
Random forest classifier	A2	0.043	0.697	1.000	0.022	1.000
	B2	0.747	0.828	0.685	0.822	0.830
Logistic regression	A2	0.333	0.752	1.000	0.200	1.000
	B2	0.677	0.724	0.532	0.933	0.630
Naive Bayes classifier	A2	0.851	0.910	0.881	0.822	0.949
	B2	0.800	0.848	0.677	0.978	0.790

Feature set A2: Features from feature set A1 retained after MI + LASSO dimensionality reduction. Feature set B2: Features from feature set B1 retained after MI + LASSO dimensionality reduction.

These results confirm that RMs could be applied across species, and screening interspecies reproducible features via *t*-test + LASSO significantly improves the practical performance of models (especially sensitivity for degenerative IVDs).

## Discussion

The accurate assessment of the degree of IVDD in experimental monkeys is crucial for further research concerning IVDD in experimental monkeys. Radiomics is an emerging field that extracts high-dimensional quantitative features from medical images, showing promising prospects in enhancing disease representation and diagnosis. When constructing RMs, a large number of datasets are needed to optimize model performance. The shortage of sample size poses significant challenges to the development of robust and reliable RMs. To this end, this study explored the generalizability of radiomics models between humans and experimental monkeys, with a focus on IVD. Here, we analyzed the reproducibility of radiomics features between humans and experimental monkey and found that a total of 12.30% (438/3562) of radiomics features were reproducible between species. Subsequently, we constructed radiomics models based on the human dataset and used the data from the experimental monkey dataset as a testing set to verify the generalizability of the model between species. In the test set, the AUCs of the models constructed based on inter species reproducible features reached 0.82–0.92. This study provides a theoretical basis for the cross-species application of radiomics.

Through RFs, it is possible to interpret disease features and understand potential pathological and physiological processes. The biological significance of RFs lies in their ability to quantitatively express macroscopic and microscopic tissue features that are invisible to the naked eye. By combining these features with advanced analytical techniques, it is possible to discover new biomarkers, improve disease classification, and guide personalized treatment strategies, ultimately promoting our understanding of disease mechanisms. The RFs extracted by PyRadiomics include First Order Statistics (first order), Shape based (3D), Shape based (2D), Gray Level Co occurrence Matrix (glcm), Gray Level Run Length Matrix (glrlm), Gray Level Size Zone Matrix (glszm), Neighboring Gray Tone Difference Matrix (ngtdm), and Gray Level Dependence Matrix (gldm).

By decomposing images into numerous RFs, radiomics could provide multi parameter characterization of IVD tissue, potentially capturing subtle changes that distinguish healthy from degenerated discs — changes that may be overlooked by traditional visual analysis. This fine-grained quantitative evaluation could enhance understanding of the key imaging features differentiating healthy and degenerated IVDs, and improve diagnostic precision for distinguishing these two states. For example, texture analysis features could reflect the characteristics of organizational microstructure. GLCM represents the statistical patterns of image texture and intensity, where features such as contrast or uniformity could provide a deeper understanding of the randomness and regularity of image grayscale. These indicators could reflect changes in cell density, fibrosis or necrosis, which are known biological indicators of disease progression or response to treatment.

Due to common sample size limitations in experimental monkey research, constructing high-precision radiomics models (RM) directly on experimental monkeys faces challenges. Therefore, we have adopted an innovative strategy: first, we use human imaging data that are sufficient for model construction, thanks to the relatively accessible sample resources in human studies. Subsequently, we attempted to apply these models to experimental monkeys to test their cross species applicability and reproducibility of radiomics features. This research design cleverly bypasses the challenge of sample size. Through comparative analysis, we aim to reveal which RFs are consistent between two species and which features may be influenced by species specificity, laying the foundation for future cross species medical research and disease understanding, and providing strong support for the development of new diagnostic and treatment methods for diseases such as intervertebral disc degeneration.

Feature reproducibility plays an important role in radiomics research^17–19^. We investigated the reproducibility of RFs between human and experimental monkey and found that a number of these features were indeed reproducible. We screened 438 features (feature set B) that were reproducible between species from 3562 features by *t*-test combined with LASSO’s method. The number of T2WI features was greater than the number of T1WI features in feature set B (255:183). In feature set B, the Image Types with the highest number of features were wavelet-HLH (50 of 438, 11.42%), and the Feature Classes with the highest number of features were glszm (125 of 438, 28.54%).

RMs constructed based on reproducible RFs could theoretically be applied across species. To verify this speculation, we used the data from the human as the training set and the experimental monkeys’ data as the test set to evaluate IVDD. In the RMs constructed based on feature sets A, and B, the AUCs in the test set were 0.70, 0.51, 0.96, 0.95, 0.96, and 0.89, 0.82, 0.88, 0.85, 0.92, respectively. This suggests that the RMs constructed based on the human’s dataset could be applied in experimental monkeys. At the same time, we also found that some RMs performed poorly in the sensitivity of identifying degenerative IVDs, with values of 0.36, 0.02, 0.02, and 0.20, respectively. However, after removing the non-reproducible features between species, the sensitivity reaches 0.82–0.96. So, the use of the *t*-test combined with the LASSO method to screen reproducible features between species could improve the performance of the model.

When screening for reproducible RFs between species using independent samples *t*-test, RFs with *p* < 0.05 could be considered to be definitely different between species. However, the opposite is not necessarily true; that is, RFs with *p* ≥ 0.05 do not necessarily differ between species. LASSO, a regression analysis method used to simultaneously perform feature selection and regularization, was first proposed by Robert Tibshirani in 1996^20^. By forcing the sum of the absolute values of the regression coefficients to be less than a fixed value, LASSO forces some regression coefficients to be zero, thus effectively selects simpler models that do not include the covariates corresponding to these regression coefficients. That is, the covariates whose regression coefficients become zero following LASSO play a smaller role in the prediction of the results. In this study, we chose covariates with regression coefficients of 0 as characteristics that are reproducible across species.

The influence of the reproducibility of RFs may be present in the various steps of radiomics, in addition to its interspecies nature^21–23^. However, compared to other steps of radiomics analysis, ROIs segmentation is often a manual and subjective process. Although automatic or semiautomatic ROIs segmentation methods are available^24^, manual segmentation of ROIs remains the gold standard; this could lead to errors when observers segment ROIs of different species. This study analyzed the effect of the two species, human and experimental monkey, on ICC, and found that experimental monkeys had a slightly lower number of features than humans with intraobserver and interobserver ICCs >0.75, except intraobserver in feature set B. Therefore, the effect of species on the ROIs segmentation needs to be considered when considering cross-species applications of RMs.

Cross-species applications of RMs generally involve data from different machine sources, so the effect of the image acquisition process is an unavoidable but necessary consideration^25^, an issue we also addressed in this study. Our results were obtained from different MRI scanners with varying acquisition parameters, which could increase feature instability. Nevertheless, the models still performed well. This underscores the robustness of the identified reproducible features, which are resilient to technical variations—a critical advantage for practical cross-species research.

Compared with previous studies, this study is innovative in comparing the reproducibility of RFs between humans and experimental monkeys, providing a theoretical basis for the cross-species application of RM. With the in-depth research and application of radiomics technology, the cross-species application of RMs has great application value. A few previous studies have used animals as subjects when generating RMs. For example, given the invasive, time-consuming, and expensive nature of lung tumor biopsy and its associated complications, Hannah Able^26^ used dogs as subjects and found that the CT RFs had prognostic utility for lung tumors. Anton S. Becker^27^ studied liver metastases using radiomics in mice before and on days 4, 8, 12, 16, and 20 after injection of MC-38 tumor cells, and the analysis revealed that textural features could quantify liver metastases. However, to our knowledge, whether these findings and RMs could be applied to humans has not previously been well established in the literature.

Our study has some limitations. First, generalization to other laboratory animals: Compared with experimental monkeys, the IVD tissue composition of other laboratory animals such as mice, rats, and rabbits, differs more from that of humans. These differences could have an impact on the reproducibility of the RFs between species. Therefore, further study is needed to better understand this reproducibility among other species in the future. Second, feature selection constraints: LASSO may arbitrarily select correlated features and saturate when features outnumber observations—a limitation mitigated here via our two-step screening process, but future studies could integrate elastic net for improved robustness. Third, Pfirrmann grading dichotomization: We simplified the 5-level Pfirrmann grading into binary categories (I-II = healthy, III-V = degenerated), which overlooks the continuous progression of IVDD and may mask early-to-moderate degeneration-related feature differences. Future studies could use ordinal classification to retain full grading details. Fourth, limited biological interpretability of radiomics features: While features effectively predict IVDD status, systematic evidence linking them to specific IVD molecular/cellular components (e.g., collagen, proteoglycans) is lacking. Future histological and molecular correlation studies will clarify their biological significance. Fifth, while this study validated the model’s performance on the entire test cohort, future research will focus on individual-level applications, such as longitudinal tracking of IVDD progression in single subjects and employing explainable AI techniques to interpret model predictions for specific individuals, thereby enhancing clinical translatability.

## Conclusion

In conclusion, this study revealed that the MRI radiomics features of intervertebral discs exhibit reproducibility across both humans and experimental monkeys, and the corresponding radiomics model could be used interchangeably between the two species. Use of the *t*-test combined with the LASSO method to screen reproducible features between species could improve the performance of the radiomics models. This study thereby furnishes a theoretical framework supporting the cross-species transferability of radiomics models, specifically between humans and experimental monkeys.

## Supplementary Information

Below is the link to the electronic supplementary material.

Supplementary Material 1

## Data Availability

The raw demographic and MRI data are protected and are not publicly available due to hospital regulations, even all the identification has been removed. Data generated or analyzed during the study are available from the corresponding author by request.

## Abbreviations

AUC: Area under the curve
ICC: Intraclass correlation coefficient
IVD: Intervertebral disc
IVDD: Intervertebral disc degeneration
LASSO: Least absolute shrinkage and selection operator
RF: Radiomics feature
RM: Radiomics model
ROC: Receiver operating characteristic
ROI: Region of interest
T1WI: T1-weighted imaging
T2WI: T2-weighted imaging

## Code availability

Some of the core code generated or used during research is available in repositories or online: https://github.com/wangjm1224/radiomics.git.
