# Supplementary material for: Cross species reproducibility of MRI radiomics features enables intervertebral disc degeneration assessment in experimental monkeys
Source: Sci Rep. 2025 Nov 28;15:45571. doi: 10.1038/s41598-025-29167-z (PMC12753628; doi:10.1038/s41598-025-29167-z)
Supplement: Supplementary file 1 — Supplementary Material 1 [file 41598_2025_29167_MOESM1_ESM.docx]

**Cross Species Reproducibility of MRI Radiomics Features Enables Intervertebral Disc Degeneration Assessment in Experimental Monkeys**

## Authors

Jianmin Wang ^a,b,1^, Lei Guo ^c,1^, Jianfeng Li ^b,1^, Xiaodong Cao ^d^, Wei Du ^a^, Jiaxiang Zhou ^b^, Haizhen Li ^b,e^, Junhong Li ^b,f^, Zhengya Zhu ^b,g^, Tao Tang ^b,h^, Xianlong Li ^b^, Zhiyu Zhou ^b,i^, Zhiguo Liu ^j,***^,Yongming Xi ^k,**^, Manman Gao ^i,l,m,*^


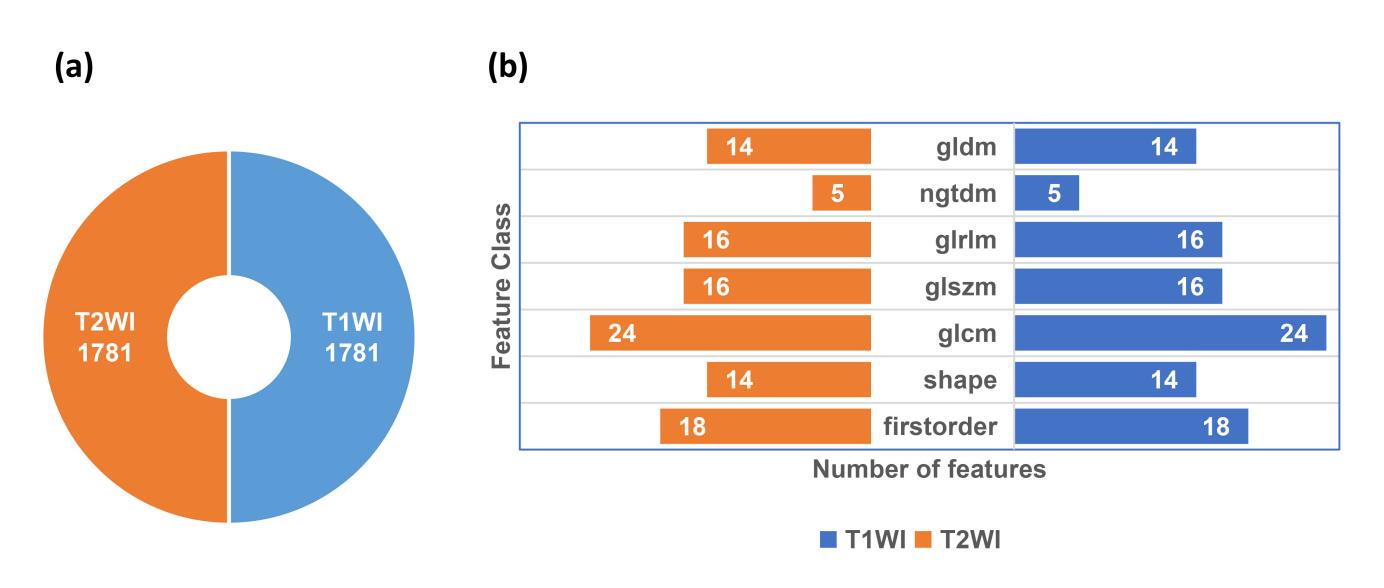


**Supplementary Fig. 1 Distribution of radiomics features.** (**a**) The same number of features was extracted from the T1WI and T2WI sequences (n=3562). (**b**) The distribution of Feature Classes on the original images (n=214). T1WI = T1-weighted imaging, T2WI = T2-weighted imaging.


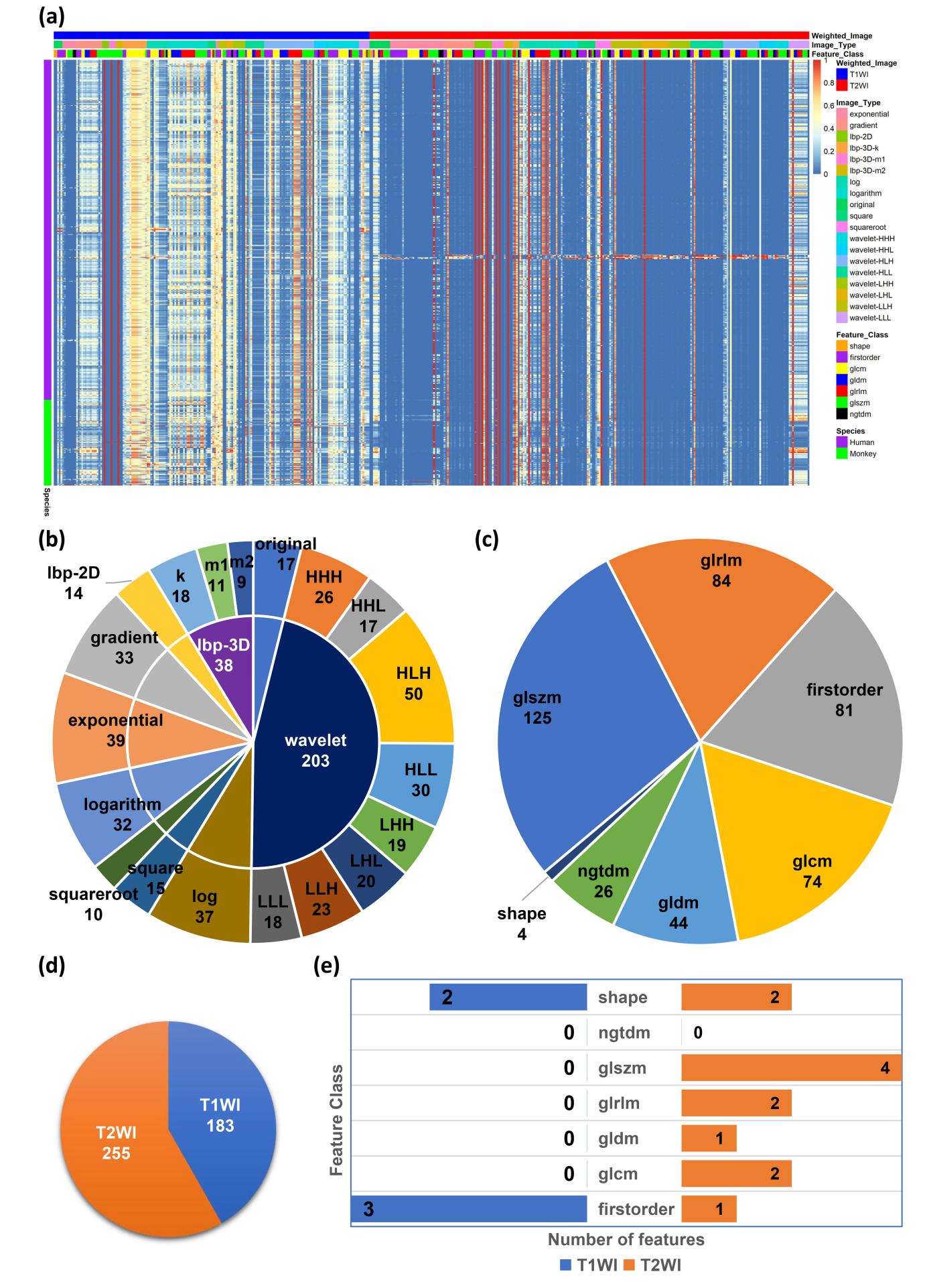


**Supplementary Fig. 2 Distribution pattern of reproducible features between species screened by t test combined with LASSO.** (**a**) Heatmap showing the 438 radiomics features that are reproducible between humans and experimental monkeys. (**b**) The feature set B is distributed across 10 major and 19 minor Image Type. (**c**) The feature set B is distributed into 7 Feature Classes. (**d**) Distribution of the feature set B for the two sequences. (**e**) The distribution of Feature Classes on the original image. n=438 in (a-d), n=17 in (e). T1WI = T1-weighted imaging, T2WI = T2-weighted imaging.


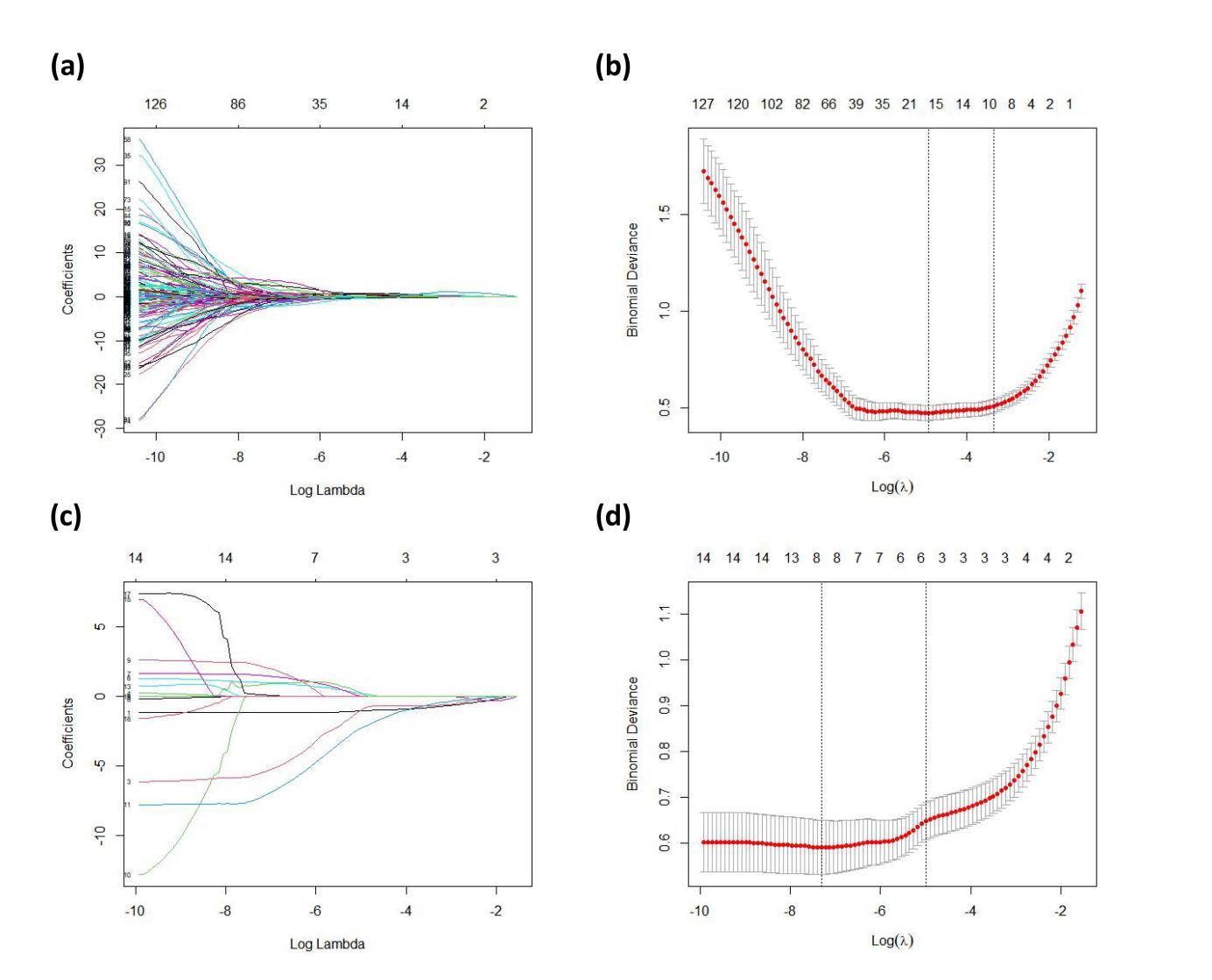


**Supplementary Fig. 3 Dimensionality reduction.** The radiomics features of feature set A1 (**a-b**), and feature set B1 (**c-d**) were screened using LASSO, and the optimal model parameters λ were selected using cross-validation. LASSO = least absolute shrinkage and selection operator.


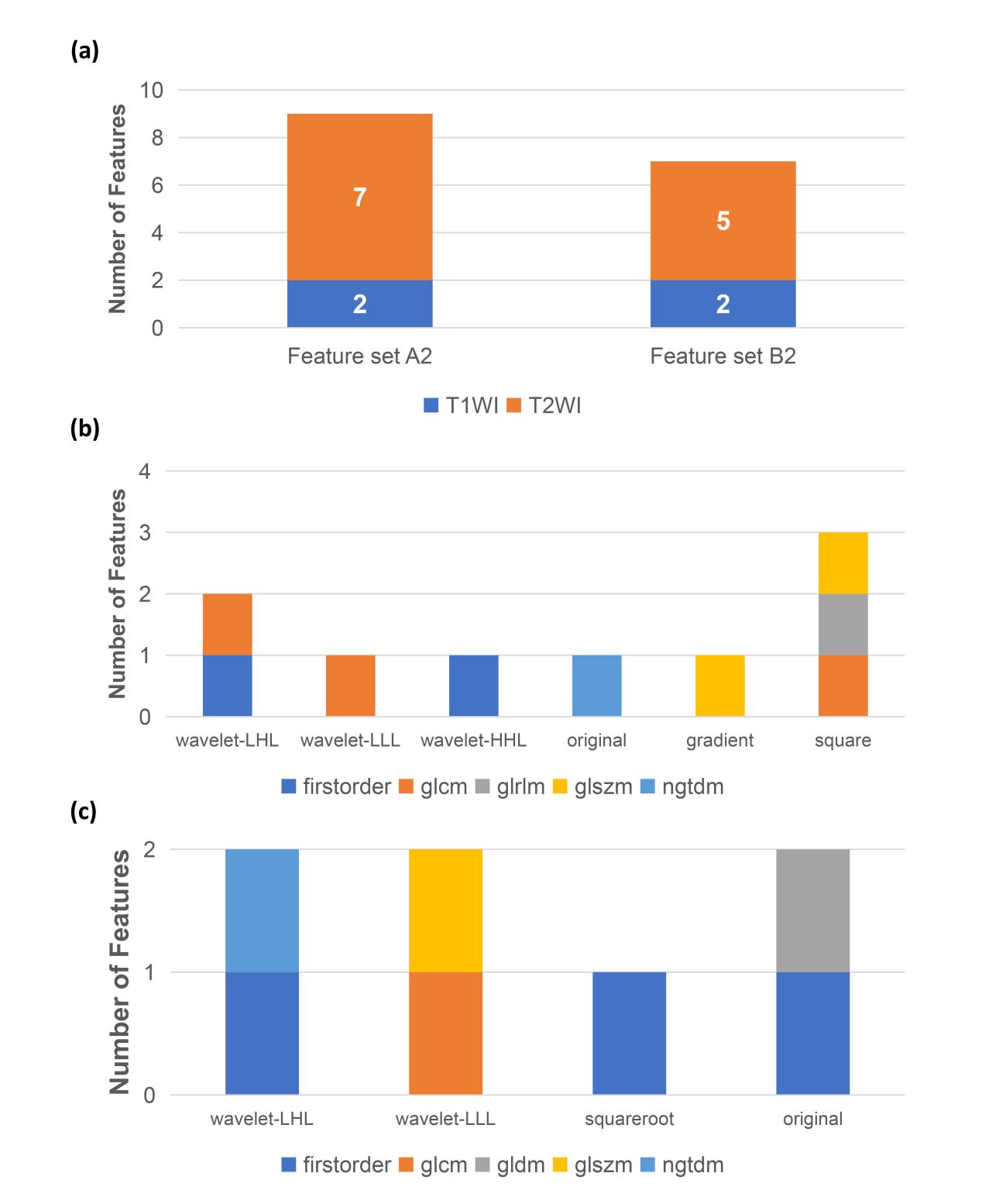


**Supplementary Fig. 4 Distribution pattern of feature set A2 and feature set B2.** (**a**) The distribution of feature set A2 and feature set B2 on T1WI and T2WI. (**b**) The distribution characteristics of feature set A2 in terms of Image Type and Feature Class. n=9. (**c**) The distribution characteristics of feature set B2 in terms of Image Type and Feature Class. n=7. T1WI = T1-weighted imaging, T2WI = T2-weighted imaging.


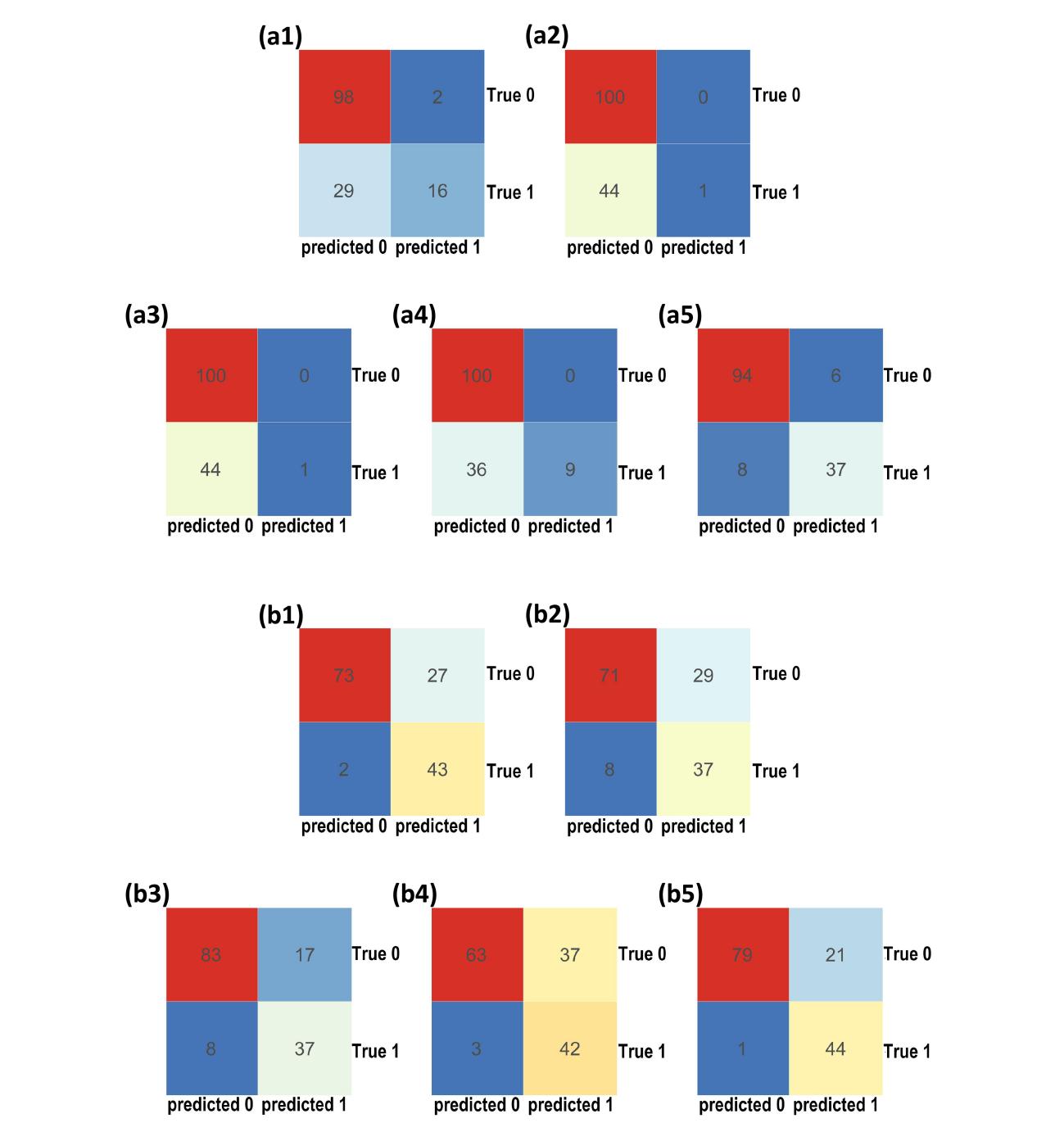


**Supplementary Fig. 5 Confusion matrix.** Confusion matrix of the radiomics models based on feature set A2 (**a**) and feature set B2 (**b**) in the test set. Model number: 1 - Support Vector Machine (SVM); 2 - Decision Tree Classifier; 3 - Random Forest Classifier; 4 - Logistic Regression; 5 - Naive Bayes Classifier. Classification of intervertebral discs: 0 - Healthy intervertebral discs; 1 - Degenerated intervertebral discs.
